# Retraction Note: A Huffman code LSB based image steganography technique using multi-level encryption and achromatic component of an image

**DOI:** 10.1038/s41598-026-52916-7

**Published:** 2026-05-12

**Authors:** Shahid Rahman, Jamal Uddin, Hameed Hussain, Aftab Ahmed, Ayaz Ali Khan, Muhammad Zakarya, Afzal Rahman, Muhammad Haleem

**Affiliations:** 1 Qurtuba University of Science and Information Technology, Peshawar, Pakistan; 2 University of Buner, Khyber Pakhtunkhwa, Pakistan; 3 Abdul Wali Khan University, Mardan, Pakistan; 4 University of Lakki Marwat, Khyber Pakhtunkhwa, Pakistan; 5 Sohar University, Sohar, Sultanate of Oman; 6 Kardan University, Kabul, Afghanistan

Retraction of: *Scientific Reports* 10.1038/s41598-023-41303-1, published online 30 August 2023

The Editors have retracted this Article.

After publication of the Article, concerns were raised about the use of non-standard phrases. Upon request, the Authors could not provide a satisfactory response to these concerns. In addition, an investigation by the Editors determined that the authorship of this Article could not be fully verified. The Editors therefore no longer have confidence in the reliability of the content of this Article.

All Authors disagree with the retraction.

